# Data article on the effect of work engagement strategies on faculty staff behavioural outcomes in private universities

**DOI:** 10.1016/j.dib.2018.04.035

**Published:** 2018-04-18

**Authors:** Hezekiah Olubusayo Falola, Maxwell Ayodele Olokundun, Odunayo Paul Salau, Olumuyiwa Akinrole Oludayo, Ayodotun Stephen Ibidunni

**Affiliations:** Department of Business Management, Covenant University, Ota, Ogun State, Nigeria

**Keywords:** Employee engagement, Career opportunities, Recognition of efforts, Job satisfaction, Fun at work, Structural equation modelling

## Abstract

The main objective of this study was to present a data article that investigate the effect of work engagement strategies on faculty behavioural outcomes. Few studies analyse how work engagement strategies could help in driving standard work behaviour particularly in higher institutions. In an attempt to bridge this gap, this study was carried out using descriptive research method and Structural Equation Model (AMOS 22) for the analysis of four hundred and forty one (441) valid questionnaire which were completed by the faculty members of the six selected private universities in Nigeria using stratified and simple random sampling techniques. Factor model which shows high-reliability and good fit was generated, while construct validity was provided through convergent and discriminant analyses.

**Specifications table**

**Table:** 

Subject area	*Human Resource Management*
More specific subject area	*Employee Engagement Strategy*
Type of data	*Table, figure*
How data was acquired	*The data were generated through structured questionnaire*
Data format	*Raw, analysed, descriptive and statistical data*
Experimental factors	*Samples consist of faculty members of the outstanding six private universities as ranked by different ranking agencies.*
Experimental features	*Work engagement strategy is a fundamental factor for building good employees disposition and behaviour in the word of work*
Data source location	*Private Universities, Southwest, Nigeria*
Data accessibility	*Data is included in this article*

**Value of the data**

• These data present information on work engagement strategies as it relates to faculty behavioural outcomes in the university context.
• Universities management can leverage on the data for decision making purposes, particularly on issues relating to work engagement strategies.
• The data can be used to identify the most predictors of work engagement strategies that will stimulate positive behavioural outcomes of the faculty members.
• The data will provide insights into what the management of the universities can do to drive productive work engagement.
• The data can be used as a platform for further investigation

## 1 Data

**Fig. 1:**
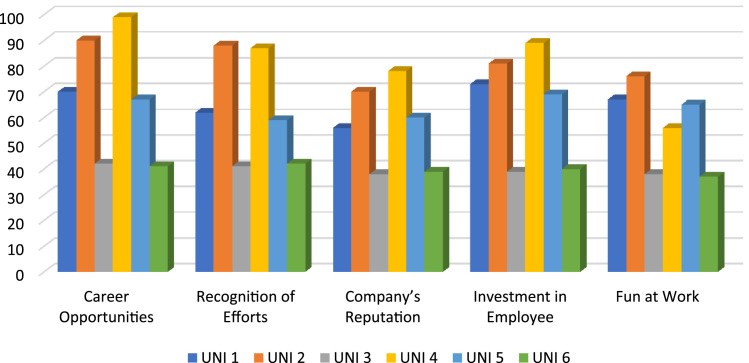
Work engagement strategies.

**Fig. 2:**
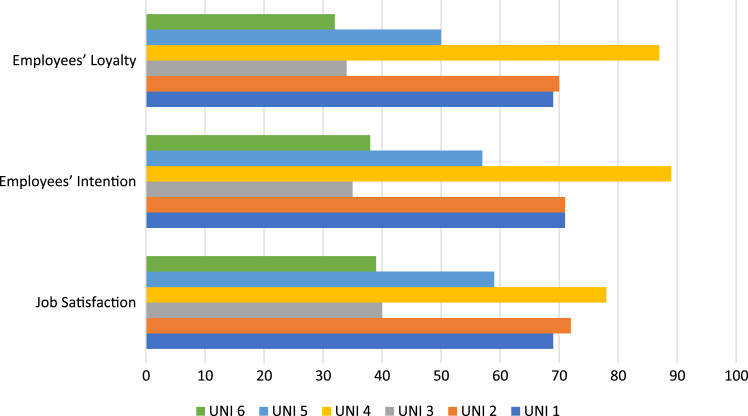
Faculty behavioural outcomes.

**Fig. 3:**
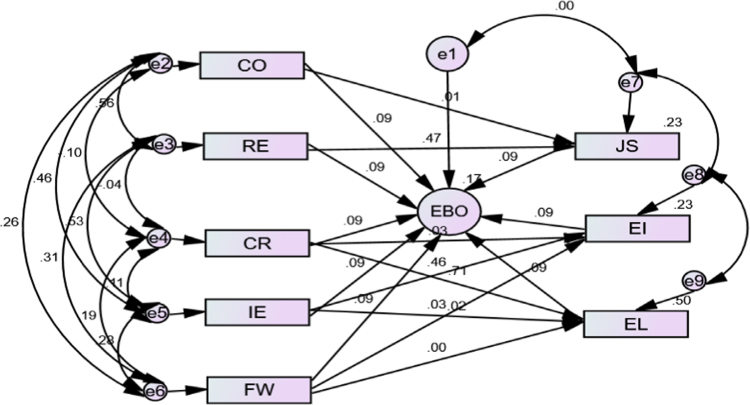
Work engagement strategies and employee behavioural outcome model.

The data presented contained raw inferential statistical on the influence of work engagement strategies on faculty behavioural outcomes. Structural equation modelling which combines factor analysis and multiple regression was used to test the structural relationships between work engagement strategies and faculty behavioural outcomes. Table 1 shows the result of validity and reliability and Table 2 shows the degree of fitness of the variables as against the minimum benchmark. In addition, the level at which respondents agreed to the measurement of work engagement strategies and faculty behavioural outcomes of each university is depicted in Fig. 1, Fig. 2. Meanwhile, the regression weight and the structural equation model depicted in Table 3 and Fig. 3 shows the model summary of the analysis. It is also important to note here that 5-point Likert scale was used for the collection of the data from the selected universities to determine the respondents’ views on the influence of work engagement strategies and faculty behavioural outcomes. The need for improved engagement in universities has become generally accepted and this depends on efficient and effective engagement of employees in corporate activities. Based on the foregoing, the data presented in this article becomes significant for investigation. However, if the data is analysed, it can help the management of the universities to have deep insight into what can be done to enhance faculty work engagement. It is also imperative to report that researchers sought for the permission of the management of the selected universities before administration of the questionnaire to the faculty members. Respondents were adequately and properly informed about the purpose of the study. They were equally given the opportunity to stay anonymous and their responses were treated with topmost confidentially.

**Table 1:** Result of validity and reliability.

		Loading	Indicator reliability	Error variance	Compose reliability	AVE	No. of final indicators
	**Variables**	**≥0.7**		**≤0.5**	**≥0.8**	**≤0.5**	
JES	Career Opportunities	0.9886	0.9773	0.0227	0.9773	0.9167	6
	Recognition of Efforts	0.8989	0.8080	0.1920	0.8080		6
	Company's Reputation	0.8953	0.8016	0.1984	0.8015		5
	Investment in Employee	0.8987	0.8077	0.1923	0.8077		5
	Fun at Work	0.8943	0. 8071	0.1929	0.8071		6
EBO	Job Satisfaction	0.9876	0.9793	0.0500	0.9500	0.9391	6
	Employees’ Intention	0.8968	0.8043	0.1957	0.8043		5
	Employees’ Loyalty	0.9284	0.8619	0.1381	0.8619		5

All loadings are significant at *p*<0.0001.

**Table 2:** Fit indices.

Indicators	GFI	AGFI	CFI	NFI	IFI	Chisq	RMSEA
Benchmark	(<0.90)	(>0.90)	(>0.90)	(>0.90)	(>0.90)	(*p*>0.05)	(<0.80)
Result	0.941	0.933	0.0903	0.988	0.908	41.173	0.5623

**Table 3:** Regression weights.

			Estimate	S.E.	C.R.	*P*
JS	<---	CO	0.006	0.087	0.064	0.949
JS	<---	RE	0.472	0.113	5.400	***
EL	<---	CR	0.705	0.060	11.970	***
EL	<---	IE	0.023	0.064	0.390	0.697
EI	<---	IE	0.464	0.086	5.921	***
EI	<---	CR	0.030	0.078	0.398	0.691
EI	<---	FW	0.027	0.080	0.353	0.724
EL	<---	FW	0.004	0.062	0.061	0.951

Note: C.R.=Critical Ratio; S.E.=Standard Error; * significant at 0.05.

The measurement model that is very paramount is the path significance indicated by the standardised regression estimate (*β*) which measures the effects of independent variable on dependent variable. In order to determine the model fit of the variables, several fit indices which include: chi-square/degree of freedom (*χ*^2^/df), Goodness-of-Fit Index (GFI) Comparative Fit Index (CFI), and Root Mean Square Error of Approximation (RMSEA) as suggested by [1], [3], [4] were examined and the output is depicted in Table 2.

Following from Table 3 and Fig. 1, the regression weight between career opportunities, recognition of effort, institutions reputation, investment on employee and fun at work in the prediction of faculty members behavioural outcome show the path coefficient of 0.006 (*p*<0.001), 0.472 (*p*<0.001), 0.905 (*p*<0.001), 0.023 (*p*<0.001) and 027 (*p*<0.05) respectively.

## 2 Experimental design, materials and methods

Data for the study were obtained through the use of self-structured questionnaire and adapted items from the reviewed literature. The 5-point Likert scale that described the extent to which the respondents agreed to the statements on the research instrument was used. The choice of the 5-point Likert scale was based on it typicality as established by scholars [2], [6]. Data were also studied and the assumptions for analysis were checked based on the procedures recommended by [1]. It was discovered that data presented were precise and accurate with no inconsistencies in various measures. It must also be noted that tolerance values were above 0.2 and variance inflation factor values were less than 5.0. Meanwhile, the analyses of normality and linearity were conducted while 159 individuals from the original sample 600 were deleted with the use of Mahalanobis distance criterion. It is equally important to report that the percentage of missing data was far less than 5 percent and this were excluded by adoption of Listwise Deletion method as suggested by some scholars [5], [7]. The final sample for the study was four hundred and forty one (441) respondents which can also be considered accurate for the analysis. After the modification of the final measurement model for all constructs, unidimensionality, reliability, and validity were evaluated and the outcomes measurement model are depicted in Table 1, summarizes the factor loadings, indicator reliability, error variance, compose reliability and average variance extracted estimate for the final measurement model.
