# Supplementary material for: Data article on the effect of work engagement strategies on faculty staff behavioural outcomes in private universities
Source: Data Brief. 2018 Apr 18;18:1383–7. doi: 10.1016/j.dib.2018.04.035 (PMC5997958; doi:10.1016/j.dib.2018.04.035)
Supplement: Supplementary file 1 — Supplementary material [file mmc1.doc]

**Falola Hezekiah Olubusayo (Ph.D)**

Department of Business Management

College of Business and Social Sciences,

Covenant University, Ota, Ogun State, Nigeria

[hezekiah.falola@covenantuniversity.edu.ng](mailto:hezekiah.falola@covenantuniversity.edu.ng)

+234 703 5518 559

**April 5, 2017**

The Editor,

Data In Brief

Dear Sir,

**DECLARATION OF CONFLICT OF INTEREST**

I, Dr. Falola H.O and my colleagues write to declare that there is no conflict of interest traceable to our data paper “Data Article on the effect of Work Engagement Strategies on Faculty Staff Behavioural Outcomes in Private Universities”

”

Thank you.

Yours faithfully,


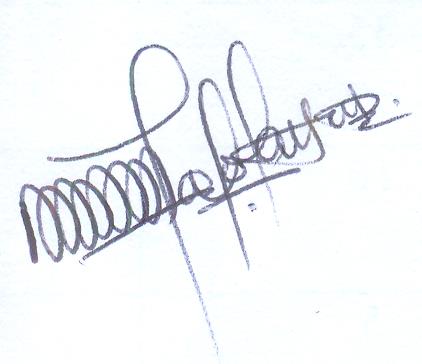


FALOLA H.O (PhD)

**Corresponding Author**
